# The evolution and application of multi-omic analysis for pituitary neuroendocrine tumors

**DOI:** 10.3389/fmed.2025.1629621

**Published:** 2025-09-01

**Authors:** Sangami Pugazenthi, Shree S. Pari, Ziyan Zhang, Julie Silverstein, Albert H. Kim, Bhuvic Patel

**Affiliations:** ^1^Taylor Family Department of Neurological Surgery, Washington University School of Medicine, St. Louis, MO, United States; ^2^Department of Neurological Surgery, University of Pittsburgh Medical Center, Pittsburgh, PA, United States; ^3^Division of Endocrinology, Metabolism and Lipid Research, Washington University School of Medicine, St. Louis, MO, United States; ^4^WashU Medicine Pituitary Center, Washington University School of Medicine, St. Louis, MO, United States; ^5^The Brain Tumor Center, Siteman Cancer Center, Washington University School of Medicine, St. Louis, MO, United States

**Keywords:** pituitary, PitNET, multiomics, molecular sequencing, transcriptomics, genomics, epigenomics, proteomics

## Abstract

Pituitary neuroendocrine tumors (PitNETs) are a heterogeneous group of intracranial neoplasms that vary in hormonal activity, histological features, and clinical behavior. The rise of high-throughput sequencing and molecular profiling technologies has enabled multiomic approaches—including genomics, transcriptomics, epigenomics, proteomics, and metabolomics—to deepen our understanding of PitNET pathogenesis. These studies have identified key mutations, transcriptional lineages, epigenetic modifications, and proteomic features that contribute to tumor subtype classification, invasiveness, and treatment response. Integrative multi-omic analyses have further revealed distinct molecular subtypes, complex regulatory networks, and molecular profiles that can predict recurrence and therapeutic efficacy. These approaches hold strong potential for advancing personalized medicine in PitNETs, supporting patient-specific diagnosis, prognostication, and therapeutic strategies. Future directions include the application of emerging -omic technologies and the development of robust computational tools to integrate and translate multi-layered data into clinically actionable insights.

## Introduction

**Figure 1:**
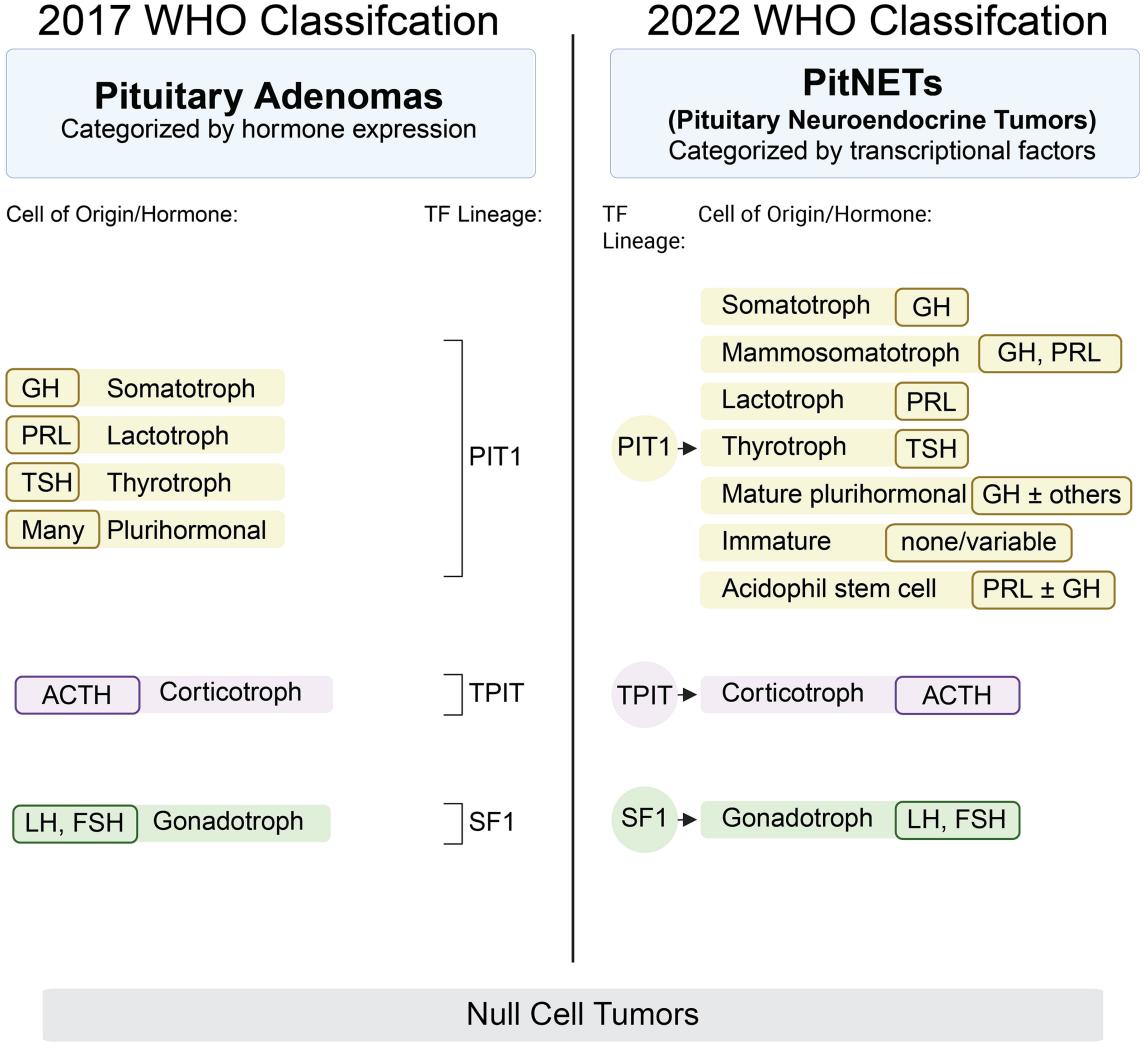
Comparison of 2017 and 2022 PitNET WHO classification schemes. PitNET, Pituitary neuroendocrine tumor; WHO, World Health Organization.

Pituitary tumors represent a diverse group of neoplasms that originate from the endocrine cells of the pituitary gland and account for approximately 17.8% of all intracranial tumors (1). Historically termed *pituitary adenomas*, these tumors have been considered largely benign and indolent. However, this perception has evolved significantly with advances in molecular pathology and clinical characterization. In 2022, the World Health Organization (WHO) officially reclassified these tumors as *pituitary neuroendocrine tumors* (PitNETs) to better reflect their neuroendocrine origin and biological spectrum (2). A pivotal aspect of the new WHO classification is the use of pituitary-specific transcription factors (TFs) to define tumor lineage more accurately than traditional hormonal immunostaining alone. The key TFs include pituitary-specific positive transcription factor 1 (*PIT1*), steroidogenic factor 1 (*SF1*), and T box transcription factor (*TPIT*), which correspond to the somatotroph/lactotroph/thyrotroph, gonadotroph, and corticotroph lineages, respectively (Figure 1). This molecular stratification helps distinguish morphologically similar but biologically distinct subtypes, thereby enhancing diagnostic precision and prognostic estimation (2). Despite these advances, the clinical management of PitNETs remains challenging due to a lack of robust biomarkers for tumor aggressiveness, treatment response, and recurrence risk. In this context, multiomic approaches—including genomic, transcriptomic, epigenomic, proteomic, and metabolomic profiling—offer powerful tools to dissect the complexity of PitNETs. Integrative multiomic analysis can provide a systems-level understanding of tumor biology, identify molecular subgroups, and uncover novel targets for therapy and early detection (3). This review summarizes the current landscape and emerging insights from multiomic studies in PitNETs, emphasizing their potential to revolutionize classification, prognosis, and individualized treatment strategies in pituitary tumor management.

## Genomic analysis

Genomic analyses have played a crucial role in uncovering the molecular underpinnings of PitNETs, shedding light on both sporadic and hereditary forms. Although PitNETs display a relatively low mutational burden compared to other solid tumors, several recurrent somatic and germline alterations have been identified that contribute to tumor initiation, hormonal dysregulation, and progression.

The most well-characterized somatic mutations in PitNETs are subtype specific. Guanine nucleotide-binding protein, alpha stimulating (*GNAS*) mutations are frequently found in somatotroph tumors, promoting cyclic adenosine monophosphate (*cAMP*) signaling and growth hormone (GH) overproduction. PitNETs with *GNAS* mutations have been associated with smaller size and decreased invasiveness (4). In corticotroph tumors causing Cushing’s Disease, ubiquitin carboxyl-terminal hydrolase 8 (*USP8*) mutations are present in up to 40% of cases and result in impaired degradation of epidermal growth factor receptor (*EGFR*), enhancing adrenocorticotropic hormone (ACTH) secretion and cellular proliferation (5, 6). Other mutations described in corticotroph PitNETs include ubiquitin specific peptidase 48 (*USP48*), B-Raf proto-oncogene, serine/threonine kinase (*BRAF*), and tumor protein p53 (*TP53*) (7, 8). Despite these discoveries, most PitNETs lack recurrent driver mutations, suggesting a significant role for epigenetic regulation, chromosomal instability, and post-transcriptional mechanisms in tumor biology. A subset of PitNETs arise in the context of hereditary tumor syndromes, most notably Multiple Endocrine Neoplasia type 1 (MEN1), caused by inactivating mutations in the *MEN1* gene, which encodes the tumor suppressor menin. Other inherited mutations involve cyclin-dependent kinase inhibitor 1B (*CDKN1B*) (associated with *MEN4*), aryl hydrocarbon receptor interacting protein (*AIP*), and succinate dehydrogenase (*SDHx*) (9–12). Patients harboring AIP mutations most commonly present with somatotropinomas, often at a younger age, with larger tumors and more growth hormone (GH) secretion (13, 14). Succinate dehydrogenase complex iron sulfur subunit B/D (*SDHB/D*) mutations have been shown to be associated with combined paragangliomas, pheochromocytomas, and less frequently PitNETs, suggesting shared tumorigenesis pathways related to mitochondrial metabolism (12).

With the development of next-generation sequencing (NGS), including whole-exome sequencing (WES) and whole-genome sequencing (WGS), studies utilizing these methods provided a broader landscape of mutational events in PitNETs. In 2016, Song et al. examined the somatic mutational landscape of 125 PitNETs, identifying low mutational burden, confirming the presence of previously described mutations such as *GNAS*, *MEN1*, and *USP8*, identifying novel mutations such as kinesin heavy chain isoform 5A (*KIF5A*) and growth factor receptor-bound protein 10 (*GRB10*), and determining that 18% of tumors harbor copy number alterations (CNAs). Gene ontology analysis revealed that plurihormonal, GH-, prolactin (PR)-, and ACTH-secreting PitNETs were enriched for somatic mutations in overlapping molecular pathways as were TSH- and LH/FSH-secreting PitNETs (15). Subsequently, Bi et al. identified that 29% of PitNETs have CNAs, but novel somatic alterations in genes were infrequent and often non-recurrent. They found that the tumors with more disrupted genomes (higher CNA burden) were more likely to be functional PitNETs or null cell tumors compared to PitNETs with less disrupted genomes, which were more likely nonfunctional (16). Large-scale sequencing efforts continue to uncover novel candidate genes and low-frequency variants that may contribute to tumor biology but integration of genomic data with transcriptomic and epigenomic profiles is essential to elucidate the mechanistic impact of these mutations, and inclusion of phenotypic data is critical for clinical relevance.

To facilitate clinical interpretation, Table 1 summarizes key PitNET biomarkers identified across multiomic studies, specifically highlighting their functional roles, prognostic value, and therapeutic relevance. Even though many markers remain investigational, this framework may inform future biomarker-guided therapy trials.

**Table 1:** Known biomarkers prognostic/therapeutic utility.

Biomarker	Subtype(s)	Alteration type	Functional role	Prognostic relevance	Therapeutic significance
GNAS	Somatotroph	Activating mutation	↑ cAMP signaling → GH hypersecretion	Smaller, less invasive tumors	Somatostatin analog sensitivity
USP8	Corticotroph	Gain-of-function mutation	↑ EGFR stability → ↑ ACTH secretion	Less aggressive, lower recurrence	EGFR inhibitors (experimental)
SF3B1	Lactotroph	Spliceosome mutation	Aberrant mRNA splicing	Potentially linked to aggressiveness	Still being investigated
HMGA2	Lactotroph	Overexpression/epigenetic activation	Chromatin remodeling	Associated with invasiveness	HDAC inhibitors (preclinical)
TERT methylation	Multiple	Promoter methylation	Telomerase activation	Conflicting; may indicate poor prognosis	Still being investigated
ID2	Corticotroph, Lactotroph	Protein overexpression	EMT regulation	Linked to invasiveness	Potential EMT targeting (preclinical)

## Transcriptional profiling

Transcriptomic profiling using techniques such as bulk and single-cell RNA sequencing has emerged as a powerful approach to characterize PitNETs beyond histology and hormonal output, offering insights into their functional identity, heterogeneity, and aggressiveness. Unlike genomic alterations, which are relatively infrequent in PitNETs, transcriptional changes are widespread and reflect both lineage commitment and tumor behavior.

Transcriptomic profiling has had a significant impact on the field of pituitary tumors as this method was used to discover the relevance of TFs in the classification of PitNETs highlighted in the 2022 WHO guidelines. The use of transcription factors has been shown to be more reliable than previous methods using histology, immunochemistry, *in situ* hybridization, and hormone expression to identify and classify these tumors (2). The biological role of *PIT1*, *SF1* and *TPIT* in normal pituitary gland development and PitNET pathogenesis has also been investigated using bulk RNA sequencing (17, 18). In normal corticotroph development, *TPIT* along with paired like homeodomain 1 (*PITX1*) activate the proopiomelanocortin (*POMC*) gene (19, 20). On the other hand, suppression of *TPIT* causes pituitary neuroendocrine cells to differentiate into gonadotroph or thyrotroph cells (21). The *PIT1* TF lineage is positively regulated by paired-like homeobox 1 (*PROP1*) and negatively regulated by HESX homeobox 1 (*HESX1*) (22, 23). Each hormonal subtype of *PIT1* PitNETs have specific mechanisms through which *PIT1* is involved in pathogenesis. Gonadotrophs are part of the *SF1*-lineage of PitNETs; *SF1* transcription in part relies on the binding of estrogen-to-estrogen receptor alpha, which mediates chromatin remodeling of the *SF1* locus (24).

Invasive PitNETs have significant differences in their transcriptional profiles compared to noninvasive tumors, including differentially expressed genes related to the Nuclear Factor-kappa B (*NF-κB*) and antitumoral immune response (25, 26). Invasive prolactinomas exhibited significantly different transcriptional profiles compared to noninvasive prolactinomas (27). Compared to noninvasive corticotrophs, invasive corticotroph tumors exhibit upregulation of cyclin D2 (*CCND2*) and zinc finger protein 676 (*ZNF676*) and downregulation of death-associated protein kinase 1 (*DAPK1*) and tissue inhibitor of metalloproteinase 2 (*TIMP2*) (28). Additionally, in corticotroph tumors, RNA-sequencing showed a decrease in RNA expression of secreted frizzled-related protein 2 (*SFRP2*), which may promote tumorigenesis by upregulating *Wnt* signaling (29). The transcriptional profile of lactotroph tumors showed activation of estrogen receptor signaling, oxidative phosphorylation signaling, and eukaryotic translation initiation factor (*EIF*) signaling. Network analysis of upstream regulators determined that potential pathogenic drivers may include early growth response 1 (*EGR1*), protein kinase cAMP-activated catalytic subunit Alpha (*PRKACA*), paired like homeodomain 2 (*PITX2*), cAMP responsive element binding protein 1 (*CREB1*), and Jun D (*JUND*) proto-oncogene, an AP-1 transcription factor subunit (30).

In addition to evaluating specific genes, pathways, and PitNET types, transcriptomic data has been used to cluster PitNETs based on molecular subtype. Consensus clustering of transcriptomic data from 117 PitNETs of all hormonal subtypes revealed three molecular subtypes of tumors defined by biological processes: Group I – signaling pathways, Group II – metabolic processes, and Group III – immune responses. Each group had different immune profiles, and Group III had the worst prognosis even though these tumors were smaller (31). Future investigation of the role of non-coding, long non-coding, micro, and circulating RNAs in PitNET biology represents a new frontier for transcriptional profiling of PitNETs (32).

Single cell RNA sequencing (scRNA-seq) has also been used to investigate biological pathways related to invasive PitNETs. Previous work has shown that silent corticotroph PitNETs have been associated with an invasive phenotype; scRNA-seq revealed that these tumors express epithelial to mesenchymal transition genes, which may be driving tumor invasion (20). scRNA-seq has also been utilized to more robustly identify the heterogeneous biology of PitNETs. For example, when analyzing tumor cells from *PIT1*-lineage tumors, expression of hormone-encoding genes represented the majority of variation between tumors. There were four major clusters of non-*PIT-1* tumor cells, and of the three clusters with majority *TPIT*-lineage tumor cells, one had significantly elevated Granzyme K (*GZMK*) expression, suggesting a possible novel subtype of corticotroph tumor. The fourth cluster of non-*PIT-1* tumor cells was predominantly composed of *SF-1* lineage cells with overexpression of follicle stimulating hormone subunit beta (*FSHB*). Additionally, within the tumor microenvironment, two distinct tumor-associated macrophage (TAM) clusters were enriched in PitNETs, one with pro-inflammatory M1 features and the other with immunosuppressive M2 marker upregulation (*SPP1*, *TREM2*, and *CX3CR1*). This finding suggests that depletion of TAMs or macrophage repolarization may be therapeutically relevant in PitNET treatment. In addition, stress response pathways were upregulated in T cells, suggesting functional exhaustion. This finding suggests that certain PitNET subtypes may be responsive to immune checkpoint blockade and other relevant tumor microenvironment modulating therapies (33).

Through the integration of scRNA-seq and single cell genomic sequencing, transcriptional profiles of normal endocrine cells (gonadotrophs, somatotrophs, and lactotrophs) to cognate tumor cells revealed several tumor-related genes such as adhesion molecule with Ig like domain 2 (*AMIGO2*), zinc finger protein 36 (*ZFP36*), BTG anti-proliferation factor 1 (*BTG1*), and disks large MAGUK scaffold protein 5 (*DLG5*) (34). Although 62% of tumors harbored CNAs, there was no significant intratumoral CNA heterogeneity (34). Although single cell molecular analyses have been utilized extensively to reveal the underlying biology and microenvironment of several cancer types and central nervous system tumors, there are only a few robust studies analyzing PitNETs at a single cell resolution. Further work in this area will likely lead to a more sophisticated understanding of PitNET tumorigenesis, especially with regard to differences between hormonal subtypes, tumor microenvironment, the immune landscape, and molecular drivers.

## Epigenetic profiling

While genomic mutations in PitNETs are relatively uncommon, epigenetic dysregulation influencing gene expression, hormonal activity, and tumor behavior has emerged as a critical mechanism of PitNET pathogenesis (35). Epigenetic changes—such as in DNA methylation, histone modifications, and chromatin remodeling—are key modulators of transcriptional activity and cellular identity in both normal pituitary cells and tumors (35). Indeed, the activity of lineage-specific transcription factors such as *PIT1*, *SF1*, and *TPIT* is modulated by epigenetic marks, and clustering of PitNETs profiled by methylation array separated tumors by TF lineage (36).

Many studies have reported epigenetic changes in numerous genes associated with cell growth, cell signaling, and cell cycle signaling, including cyclin dependent kinase 1 (*CDK1*), cyclin dependent kinase inhibitor 1B (*CDKN1B*), cyclin dependent kinase inhibitor 2A (*CDKN2A*), cyclin dependent kinase inhibitor 2C (*CDKN2C*), growth arrest and DNA damage inducible gamma (*GADD45G*), Ras association domain family member 1 (*RASSF1A*), Ras association domain family member 3 (*RASSF3*), *DAPK*, pituitary tumor transforming gene 1 (*PTTG1*), maternally expressed 3 (*MEG3*), and fibroblast growth factor receptor 2 (*FGFR2*) (37–51). More aggressive PitNETs, defined by larger size and invasiveness, have been associated with the overexpression of DNA methyltransferases 1/3A (*DNMT1/*3A) and promoter hypermethylation of tumor suppressor genes (52). The first genome-wide methylation analysis of PitNETs in 2012 identified differentially methylated genes in nonfunctioning, GH-, and PRL-secreting PitNETs. Specifically, HHIP like 1 (*HHIPL1*) and transcription factor AP-2 epsilon (*TFAP2E*) were hypermethylated in nonfunctioning tumors (53). Multiple studies have shown that these nonfunctional tumors have global hypermethylation compared to hormonally active tumors (53–55). However, *invasive* nonfunctioning tumors have more hypomethylated cytosine-phosphate guanine (CpGs) sites compared to *noninvasive* nonfunctioning tumors (54), reminiscent of the global hypomethylation observed in many cancers (56). Biological pathways that were differentially methylated between invasive and noninvasive PitNETs included homophilic cell adhesion, cell–cell adhesion, and biological adhesion. The Polypeptide N-acetylgalactosaminyltransferase 9 (*GALNT9*) promoter was also found to be methylated with corresponding decreased RNA expression in invasive tumors, making *GALNT9* expression a potential therapeutic target (55).

Although telomerase reverse transcriptase (*TERT*) promoter mutation is a marker of aggressiveness in numerous cancers and central nervous system tumors, the role of *TERT* promoter alterations such as methylation has been debated in PitNETs. In 2018, a study with 101 patients found no relationship between *TERT* promoter mutation or methylation and outcomes in patients with PitNETs (57). However, in a 2019 study analyzing 70 patients, *TERT* promoter methylation was associated with disease progression and shorter progression free survival (58, 59). Other common epigenetic biomarkers in brain tumors such as glioma include O6-methylguanine-DNA methyltransferase (*MGMT*) promoter methylation, which is related to response to temozolomide (TMZ) therapy. In contrast, in PitNETs the relationship between *MGMT* methylation status and prognosis or response to TMZ remains controversial (60–63).

Despite the ongoing debate surrounding prognostic epigenetic biomarkers like *MGMT* in PitNETs, the broader role of the epigenetic machinery itself presents a compelling target for therapeutic intervention. Importantly, DNA methyltransferase (DNMT) inhibitors and histone deacetylase (HDAC) inhibitors have demonstrated efficacy in other central nervous system tumors like glioblastoma and may be clinically relevant for the treatment of aggressive PitNETs (64, 65). While not yet clinically validated in PitNETs, DNMT and HDAC inhibitor therapies could be particularly beneficial when conventional therapies fail. Preclinical PitNET models will be essential in determinng whether modulation of the epigenetic landscape can suppress tumor proliferation, reduce hormonal hypersecretion, or enhance sensitivity to standard treatments such as temozolomide. As we further study PitNET epigenetics, targeted manipulation of regulators such as DNMTs and HDACs may emerge as a viable therapeutic strategy within a precision medicine framework.

## Proteomic analysis

Proteomic analysis provides a direct readout of the functional state of cells by quantifying proteins and their post-translational modifications. In PitNETs, proteomic analyses offer unique insights into tumor activity, cellular heterogeneity, and treatment response.

Advanced mass spectrometry (MS)-based techniques, including tandem MS and data-independent acquisition (DIA), have enabled high-throughput profiling of PitNET proteomes and post-translational modifications. MS analysis reveals that nonfunctioning PitNETs have 2,000–6,000 differentially expressed proteins compared to normal pituitary glands (66, 67). Proteomic methods have also been used to identify the role of phosphorylation of proteins in nonfunctioning PitNETs. For example, phosphorylation of *β*-catenin at Serine552 is associated with aggressive disease characterized by invasion and recurrence (68). Meanwhile, comparison of nonfunctioning tumors to normal pituitary glands revealed 595 differentially phosphorylated proteins associated with biological pathways such as the spliceosome pathway, RNA transport pathway, and proteoglycans in cancer (69). Ubiquitination is another post-translational modification that has been investigated in PitNET biology. Ubiquitinated proteins in PitNETs were most involved in biological pathways such as the Phosphatidylinositol 3-kinase/protein kinase B (PI3K/AKT) pathway, Hippo (Hpo) pathway, ribosome signaling pathway, and nucleotide excision repair (70).

Alterations of specific protein abundances and functions have been investigated to identify their role in tumorigenesis in PitNETs. For example, hematopoietic cell signal transducer 1 (Hint1) is a protein marker that was found to have high expression in invasive PitNETs, especially those that expressed vascular endothelial growth factor (VEGF) and fetal liver kinase 1 (Flk1) (71). Invasive tumors were also found to have higher expression of cluster of differentiation 206 (CD206), a M2-macrophage marker, compared to noninvasive tumors based on immunohistochemical staining (72). Several protein components of the Notch pathway were altered in prolactinomas, in addition to increased expression of PIT1 and survival factor phosphoprotein associated with glycosphingolipid-enriched microdomains 1 (PAG1) and decreased expression of E-cadherin and N-cadherin (73).

Nitroproteomics is a subfield of proteomics that specifically studies nitropeptides and nitroproteins, which are often markers of oxidative damage and can be associated with tumorigenesis. In studies investigating nitroproteins in PitNETs, several nitroproteins and other proteins that interact with nitroproteins in nonfunctioning PitNETs were discovered using a nitrotyrosine affinity column (NTAC) (74, 75). Analysis of nitroproteins is important since identification of post-translational modifications such as nitrosylation may suggest potential new avenues for targeted therapy (76). Further work to identify the extent of the role of nitroproteomics in PitNET biology and tumorigenesis is warranted.

## Metabolomics

Metabolomics—the comprehensive profiling of small-molecule metabolites in biological samples—provides a dynamic snapshot of cellular metabolism and its interaction with the tumor microenvironment. In PitNETs, metabolomic analysis has begun to uncover metabolic adaptations associated with hormone synthesis, tumor growth, and treatment resistance (77). Metabolomic methods such as matrix-assisted laser desorption/ionization (MALDI) mass spectrometry imaging have been used to confirm excess hormone production and classify PitNETs within 30 min (78). In patients with Cushing’s disease, biomarkers such as pyridoxate, deoxycholic acid, and 3-methyladipate were altered in plasma samples (79). Urine metabolites were analyzed using gas chromatography mass spectrometry system in prolactinoma patients, which showed an elevation of urinary 17-ketosteroids and all estrogen metabolite concentrations, as well as the ratios of delta 5/delta 4-steroids and 5 beta/5 alpha- hydrogensteroids (80). These findings have implications for understanding tumor biology, the systemic effect of disease, and identification of measurable biomarkers. For instance, PitNETs are defined by a distinct metabolic profile with higher succinic and lactic acid (72). These finding suggest possible mechanisms of disease development and progression as well as identification of biomarkers for diagnosis and targeted therapy. Although still an emerging field in pituitary tumor research, metabolomics holds significant promise for identifying biomarkers and therapeutic vulnerabilities, particularly in combination with other -omic methods.

## Integrative Multiomic analysis

**Figure 2:**
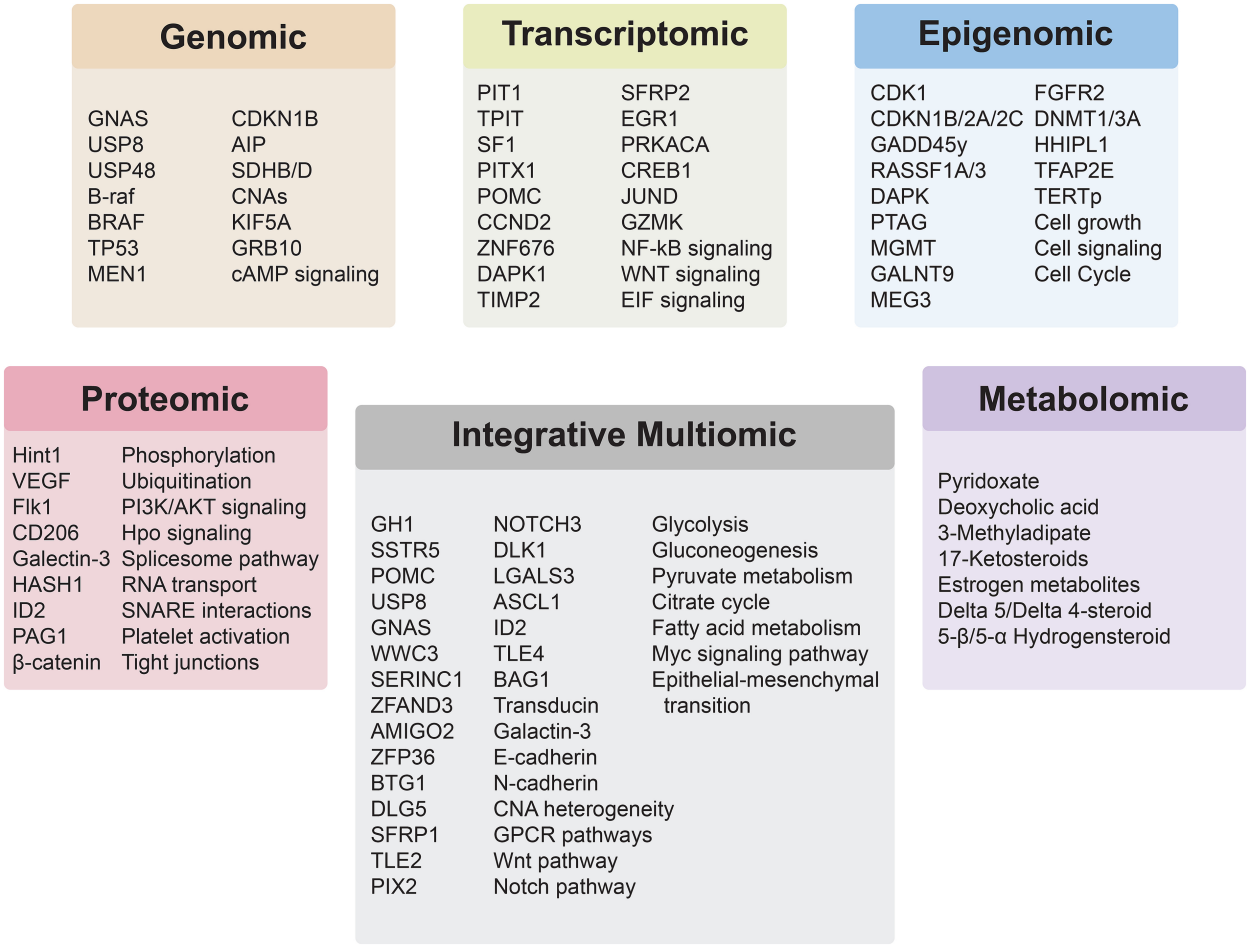
Insights derived from the application of individual and integrative multiomics analyses for PitNETs.

The advent of high-throughput -omics technologies has revolutionized our understanding of PitNETs, enabling comprehensive analyses at multiple molecular levels. These technologies each offer distinct advantages and limitations in terms of resolution, sensititivty, sample input, cost, and use-case. Table 2 provides a comparative overview of commonly used technologies across omics layers in an effort highlight pragmatic and methodological constraints across PitNET research. Integrative -omic analysis provides a holistic view of the molecular landscape of PitNETs, facilitates identification of biomarkers, elucidates complex regulatory networks, and uncovers potential therapeutic targets. Recent studies have demonstrated that such integrative analyses can reveal distinct molecular subtypes of PitNETs, improve correlations between molecular profiles and clinical outcomes, and provide insights into tumorigenesis and progression (Figure 2).

**Table 2:** Omics technology comparison table.

Omics layer	Technology	Resolution	Noise/Artifacts	Sample input	Cost	Use case in PitNETs
Genomics	Whole-Exome Sequencing (WES)	Coding regions only	Misses non-coding mutations	Low (DNA only)	Lower	Detects recurrent mutations (e.g., GNAS, USP8)
	Whole-Genome Sequencing (WGS)	Genome-wide	Higher data volume; difficult to interpret	Moderate to high	High	Detects CNAs, structural variants, non-coding mutations
Transcriptomics	Bulk RNA-seq	Average expression across all cells	Cell-type heterogeneity obscured	Moderate (bulk RNA)	Moderate	Captures bulk transcriptional signatures and TF expression
	Single-cell RNA-seq	Cell-level resolution	High dropout rate, technical variability	High quality single cells	High	Uncovers heterogeneity, subclonal expression, TME profiles
Epigenomics	Methylation Profiling	CpG-rich regions	Biased methylome coverage	Low (DNA)	Low to moderate	Differentiates TF-defined subtypes; correlates with RNA expression
Proteomics	Mass Spec based Proteomics	Protein-level, post translational	Stochastic sampling, high data volume	Moderate	Moderate to high	Identifies differentially expressed proteins and PTMs

As Table 3 summarizes, each PitNET subtype is characterized by distinct molecular features across genomic, transcriptomic, epigenomic, proteomic, and metabolomic layers. Despite their differences, these multiomic signatures converge on shared biological pathways across subtype. For instance, somatotroph tumors exhibit GNAS mutations, PIT1-driven transcription, and enrichment of proteins in PI3K/AKT signaling, which collectively support growth hormone hypersecretion via cAMP signaling and metabolic reprogramming (81). Corticotroph tumors exhibit USP8 mutations, upregulation of proopiomelanocortin (POMC), transcriptomic changes in Wnt regulators like SFRP2, and proteomic changes in Galectin-3 and ID2, linking chromatin remodeling and epithelial-to-mesenchymal (EMT) transition with sustained ACTH hypersecretion (82–84). Finally, lactotroph tumors with FIPA or SF3B1 mutations and estrogen receptor activation display epigenetic change (HMGA regulation via chromatin architecture) and proteomic shifts in Galectin-3, HADH1, and ID2, linking genetic mutations and estrogen signaling to altered tumor epigenetics and protein expression patterns that drive tumor aggressiveness and treatment resistance (85, 86). These convergences evidently highlight shared mechanisms such as hormone hypersecretion, chromatin remodeling, biological pathway activation, and metabolic rewiring across tumor types, underscoring the translational value of integrative multiomic analysis in PitNET research. Additional molecular studies across different subtypes remain necessary, as certain subtypes such as Gonadotroph PitNETs lack any published molecular data (87).

**Table 3:** Molecular features of PitNETs by hormonal expression.

Cell of origin	Somatotroph	Lactotroph	Thyrotroph	Corticotroph	Gonadotroph
Hormone	Growth Hormone	Prolactin	Thyroid stimulating hormone	Adrenocorticotrophic hormone	Luteinizing hormone/follicle stimulating hormone
Transcription Factor	PIT1	PIT1	PIT1	TPIT	SF1
% of all PitNETs	11	40	0.2	6	43
Molecular features					
Genomics	GNAS AIP	FIPA SF3B1	MEN1 AIP ASTN2 CWH43 R3HDM2 SMOX STYL3 ZSCA23 CNAs	USP8 USP48 BRAF TP53	
Transcriptomics	Three transcriptional subtypes ODC BAG1	Activation of estrogen receptor, oxidative phosphorylation, and EIF signaling		SFRP2 Wnt signaling CCND ZN DAPK1 TIMP2	
Epigenomics		HMGA regulation via chromatin architecture	SLIT1		
Proteomics	IL-4 PDGF PTEN VEGF PI3K/AKT FAK	Galectin-3 HASH1 ID2		Galectin-3 HASH1 ID2	
Metabolomics		Urine 17-ketosteroids Succinic acid Lactic acid		Pyridoxate Deoxycholic acid 3-methyladipate	
Multiomics	GH1 SSTR5 GPCR pathway ATP2A2 ARID5B WWC3 SERINC1 ZFAND3	Notch pathway E-cadherin N-cadherin	Fatty acid metabolism Nitrogen metabolism Insulin PPAR HIPPO PIP5K1B NEK10	POMC Glycolysis Gluconeogenesis Pyruvate metabolism Citrate cycle Fatty acid metabolism Myc signaling	

Although genomic profiling suggests infrequent rates of somatic mutations in PitNETs, CNAs are common among all TF-lineage subtypes. Integrating analysis of methylation and transcriptional data suggests that hypomethylation of promoter regions is associated with increased RNA expression of *GH1* and Somatostatin Receptor subtype 5 (*SSTR5*) in GH-secreting PitNETs and *POMC* in ACTH-secreting PitNETs (88). In a 2020 multi-omic study, three molecular classes of PitNETs were identified by integrating somatic mutations, chromosomal alterations, and profiling of the miRNAome, methylome, and transcriptome (89). This classification scheme clustered PitNETs similar to the classification based on TF lineage. Prognostic analysis identified that *USP8* wildtype (WT) compared to *USP8* mutant corticotroph PitNETs were more aggressive with invasive properties (89). The transcriptome of these invasive corticotrophs was enriched for genes associated with epithelial-mesenchymal-transition, consistent with their invasive clinical behavior (89). Gene ontology analysis in a transcriptomic and proteomic integrated analysis of *GNAS* mutant vs. wildtype somatotrophs suggested that *GNAS* mutations may impact endocrine features through induction of G protein-coupled receptor (GPCR) pathways. Higher protein expression of WW and C2 domain-containing protein-3 (WWC3), serine incorporator 1 (SERINC1), and zinc finger AN1-type containing 3 (ZFAND3) was correlated with increased tumor volume after somatostatin analog treatment (90). Recurrence as a clinical marker of aggressive disease has also been investigated utilizing multiomic methodologies. A robust longitudinal study of primary and recurrent PitNETs from the same patient determined primary and recurrent PitNETs to have similar genomic profiles but divergent transcriptomic profiles (91). Interestingly, several metabolic pathways that were differentially expressed among primary and recurrent tumors based on transcriptional data did not seem to be regulated by methylation, raising the possibility of alternative regulatory mechanisms that warrant further investigation (91).

Multiomic analyses have also incorporated both proteomic and transcriptomic data to further understand PitNET biology. For example, nonfunctioning PitNETs had almost 300 differentially expressed genes and 50 differentially expressed proteins compared to controls including secreted frizzled-related protein 1 (*SFRP1*), transducin like enhancer of split 2 (*TLE2*), *PITX2*, Notch receptor 3 (*NOTCH3*), and delta like non-canonical Notch ligand 1 (*DLK1*) (92). These findings suggest potential critical molecular pathways implicated in this tumor type such as the Wnt and Notch pathways. Integrative proteomic and transcriptomic analysis has also been used to analyze metastatic PitNETs, which led to the identification of almost 5,000 differentially expressed genes, and the downregulation of beta-galactoside binding protein galactin-3. Other genes that may play important roles in metastatic PitNETs include lectin, galactoside-binding, soluble, 3 (*LGALS3*), achaete-scute family bHLH transcription factor 1 (*ASCL1*), *ID2*, and transducin like enhancer of split 4 (*TLE4*) (93). Lastly, transcriptomic and proteomic analysis of prolactinomas compared to normal pituitary glands identified a unique transcriptomic and proteomic profile. Notably, several components of the Notch pathway were altered in prolactinomas, along with increased expression of survival factor BCL2 associated athanogene 1 (BAG1) and decreased expression of E-cadherin and N-cadherin (73).

Metabolomics has been used alongside other -omic methods such as proteomics and lipidomics to delve further into the mechanisms of PitNET pathogenesis. In ACTH-secreting PitNETs, integrated analysis identified that these tumors were significantly enriched in protein-metabolite joint pathways such as glycolysis/gluconeogenesis, pyruvate metabolism, citrate cycle, and fatty acid metabolism (94). The Myc signaling pathway was also identified to have a significant role in the metabolic changes and tumorigenesis of these tumors (94). A broader study using desorption electrospray ionization (DESI-MS) derived phospholipid signals that differed between gray matter, white matter, gliomas, meningiomas and pituitary tumors. Principal component analysis of lipid and metabolite profiles from this analysis were able to separate different tumor types: gliomas, meningiomas, and pituitary tumors (95).

However, while these studies underscore the value of integrative multiomics, they also highlight the significant computational hurdles in merging heterogenous omic datasets. Despite the growing number of multi-omic studies in PitNETs, integration and standardization across datasets remain computationally challenging, as omics data is inherently heterogenous. Several bioinformatic tools have been developed to address these issues. Multi-omics factor analysis uses unsupervised latent factor modeling to identify hidden sources of variation across omics layers (96). Similarity network fusion constructs networks of samples and merges these networks effectively to discover subtypes (97). By contrast, iClusterPlus applies joint latent variable modeling to integrate multiple subtypes of genomic data for subtype identification (98). Unfortunately, these distinct data fusion techniques differ in scalability, handling of missing data, and interpretability. Moreover, these methods are rarely tailored to PitNET-specific datasets, which tend to be small and sparse.

Standardization of data in PitNET omics research faces similar issues. Batch effects, inconsistent normalization strategies, and variable bioinformatics pipelines undermine reproducibility of data. Transcriptomic analysis heavily relies on normalization and batch correction tools like ComBat or Harmony (99, 100). Proteomic and epigenomic analyses use quantile normalization and reference-based scaling to address technical variability (101). Collectively, these techniques’ inconsistencies can complicate downstream integration efforts. Hence, adhering to data frameworks such as the NIH’s Findable, Accessible, Interoperable, Reusable (FAIR) principles, standardizing pipelines, and reporting metadata in PitNET research would allow for increased reproducibility and comparability of data, facilitating the development of robust PitNET-specific computational pipeline that provide clinically meaningful data.

In parallel with efforts to integrate and standardize multiomic workflows, artificial intelligence (AI) and machine learning (ML) have emerged as powerful tools for analyzing complex multi-omic datasets. Although still in the nascent stages of adoption in PitNET research, these methods are beginning to prove extremely useful. Several studies have already utilized AI and ML to create robust PitNET classifiers for risk stratification and diagnosis. Wang et al. used LASSO regression and Support Vector Machine Recursive Feature Elimination to develop a Programmed Cell Death-associated index (PCDI) classifier that outperforms traditional prognostic models in identifying invasive PitNETs with a high degree of accuracy (102). In another study, Li et al. used radiomic features derived from T2-weighted MRI to construct a Gaussian process model capable of preoperatively predicting histological subtypes of PitNETs, such as prolactinoma (103). Despite these promising results, the translational potential of these approaches is limited by the paucity of PitNET datasets. Collaborative future modeling efforts may allow for more robust and accurate model construction and generalization.

Integrative multi-omics analyses have significantly advanced our understanding of PitNETs by revealing multiple molecular subtypes and the complex regulatory networks that underlie tumor behavior. Building upon these approaches, spatial omics technologies are emerging as vital tools for resolving tumor heterogeneity in its native context. Spatial transcriptomics and proteomics offer significant resolution advancement for characterizing intratumoral heterogeneity and tumor microenvironment architecture in PitNETs. For instance, spatial transcriptomics could distinguish between non-invasive and invasive PitNET phenotypes by localizing EMT markers. Similarly, spatial proteomic analysis could enable the visualization of PTMs throughout the invasive PitNET front. These tools have the potential to refine the current understanding of PitNET pathophysiology and support the development of spatially-informed, precision medicine strategies.

## Translational gaps

While multiomics PitNET research has yielded invaluable biological insights, a significant gap remains between academic discovery and clinical translation. Cost and infrastructure requirements for generating and analyzing multi-layered omics data remains prohibitive, especially outside of academic centers. Governmental regulatory pathways for clinical grade omics assays are still evolving, with no PitNET omics-based biomarker panels still having received FDA clearance. Clinical trials for multiomic biomarker validation also remain rare and underpowered.

## Conclusion

In conclusion, the integration of multi-omics technologies has profoundly advanced our understanding of PitNETs, offering a comprehensive view of their molecular landscape. By combining data from genomics, transcriptomics, proteomics, epigenomics, and metabolomics, researchers have identified distinct molecular subtypes, unveiled regulatory networks, and discovered novel biomarkers, thereby enhancing diagnostic precision and informing therapeutic strategies. Clinically, these integrative approaches hold promise for the development of personalized medicine in PitNET management, which is a critical need, in particular for recurrent tumors and tumors not cured by the current standard of care. The ability to correlate multiomic profiles with clinical outcomes facilitates more accurate prognostication and the potential for tailored treatment regimens. Looking forward, the continued evolution of computational tools and machine learning algorithms will be critical in managing the complexity of multiomic data, enabling real-time integration and interpretation in clinical settings. Advancements in single-cell and spatial omics technologies are expected to further define tumor heterogeneity and microenvironmental interactions, providing deeper insights into PitNET pathogenesis. Collectively, these developments herald a new era in PitNET management, where multiomic integration becomes central to patient-specific diagnosis, prognosis, and therapy.
